# Combining high pressure and electric fields towards *Nannochloropsis oculata* eicosapentaenoic acid-rich extracts

**DOI:** 10.1007/s00253-023-12626-w

**Published:** 2023-06-29

**Authors:** Sérgio Sousa, Ana P. Carvalho, Carlos A. Pinto, Renata A. Amaral, Jorge A. Saraiva, Ricardo N. Pereira, António A. Vicente, Ana C. Freitas, Ana M. Gomes

**Affiliations:** 1 Universidade Católica Portuguesa, CBQF-Centro de Biotecnologia e Química Fina-Laboratório Associado, Escola Superior de Biotecnologia, Rua Diogo Botelho 1327, 4169-005 Porto, Portugal; 2 REQUIMTE/LAQV-Instituto Superior de Engenharia, Instituto Politécnico do Porto, Rua Dr. António Bernardino de Almeida, 431, 4200-072 Porto, Portugal; 3 LAQV-REQUIMTE–Department of Chemistry, University of Aveiro, 3810-193 Aveiro, Portugal; 4 CEB–Centre of Biological Engineering, University of Minho, Braga, Portugal

**Keywords:** Microalgae, Omega-3 polyunsaturated fatty acids, Osmotic stress, High hydrostatic pressure, Moderate electric fields, Solvent mixture

## Abstract

**Abstract:**

*Nannochloropsis oculata* is naturally rich in eicosapentaenoic acid (EPA). To turn this microalga into an economically viable source for commercial applications, extraction efficiency must be achieved. Pursuing this goal, emerging technologies such as high hydrostatic pressure (HHP) and moderate electric fields (MEF) were tested, aiming to increase EPA accessibility and subsequent extraction yields. The innovative approach used in this study combined these technologies and associated tailored, less hazardous different solvent mixtures (SM) with distinct polarity indexes. Although the classical Folch SM with chloroform: methanol (PI 4.4) provided the highest yield concerning total lipids (166.4 mg_lipid_/g_biomass_), diethyl ether: ethanol (PI 3.6) presented statistically higher values in terms of EPA per biomass, corresponding to 1.3-fold increase. When SM were used in HHP and MEF, neither technology independently improved EPA extraction yields, although the sequential combination of technologies did result in 62% increment in EPA extraction. Overall, the SM and extraction methodologies tested (HHP—200 MPa, 21 °C, 15 min, followed by MEF processing at 40 °C, 15 min) enabled increased EPA extraction yields from wet *N. oculata* biomass. These findings are of high relevance for the food and pharmaceutical industries, providing viable alternatives to the “classical” extraction methodologies and solvents, with increased yields and lower environmental impact.

**Key points:**

• *Et*_*2*_*O: EtOH is a less toxic and more efficient alternative to Folch solvent mixture*

• *HHP or MEF per se was not able to significantly increase EPA extraction yield*

• *Combinations of HHP and MEF technologies increased both lipids and EPA yields*

**Graphical abstract:**

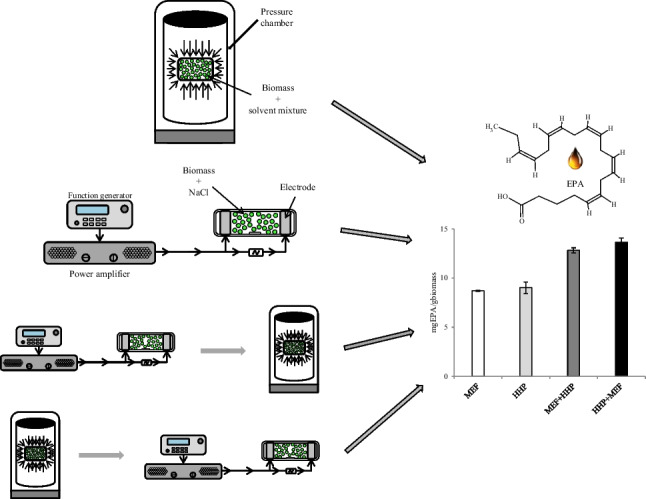

## Introduction

Microalgae are widely recognized as a potential source of distinct compounds such as pigments, vitamins, proteins, carbohydrates, and lipids. They can, therefore, be exploited by several different industries, either used “directly” as single cells, or as sources of bioactive compounds for health and pharmacological products (Parniakov et al. 2015; de Souza et al. 2019; Bueno et al. 2020).

Concerning their lipid profile, some microalgae can be regarded as an interesting source of omega-3 (ω-3) polyunsaturated fatty acids (PUFA), specifically eicosapentaenoic acid (EPA) and docosahexaenoic acid (DHA). These are known to positively impact human health, presenting beneficial effects on brain and retinal development, dementia, depression, anti-inflammatory activity, and reduction of cardiovascular diseases (Ma et al. 2016; Peltomaa et al. 2018; Udayan et al. 2018). As sources of such compounds, microalgae can be an alternative to fish oil, the most widespread commercial source (Peltomaa et al. 2018). However, in order to be an economically viable alternative, efficient extraction techniques are needed, aiming to achieve maximal yields of compounds of interest (Ramesh Kumar et al. 2019).

Traditionally, lipid extraction is performed with the use of organic solvents such as chloroform (Chl), which are detrimental to health and the environment. In this sense, there is an increasing demand for methodologies and technologies that use less hazardous solvents. This goal has driven researchers to explore new technologies to couple with alternative solvents, aiming to extract compounds with similar or higher yields than the ones obtained with traditional solvents. Ethanol (EtOH) has been studied as an alternative greener solvent (Baumgardt et al. 2016; Bueno et al. 2020; Pagels et al. 2021), but its non-selectivity for lipids narrows its range of application. Among alternative technologies, several use pressure—high hydrostatic pressure (HHP) (Khan et al. 2018), high pressure homogenization (Samarasinghe 2012), pressurized fluid extraction, and subcritical and supercritical fluid extraction (Herrero and Ibáñez 2015)—whereas others use an electric field—pulsed electric fields (PEF), high voltage electric discharge (HVED), moderate electric fields (MEF) (or alternate current; AC), and direct current (DC) (Geada et al. 2018). The aim of all these technologies is either to improve cell permeability, cause cell destruction, or increase solvent penetration, so that the solvents can easily access the compounds to be extracted, or to improve solvent selectivity, which allows the solvent to better extract a specific type of compound.

High hydrostatic pressure (HHP) has been extensively explored as a methodology to extract bioactive compounds, mainly from food products and wastes (Khan et al. 2018). Recent studies extracted flavonoids and lycopene from tomato pulp (Briones-Labarca et al. 2019), phenolics from watercress (Pinela et al. 2018), high-molecular-weight melanoidins from black garlic (Zhao et al. 2019), and ω-3 PUFA from liquid wastes of fish canning industry (Monteiro et al. 2018). However, there are few studies concerning HHP as a technology to perform extractions of lipids from microalgae (Bueno et al. 2020; Gallego et al. 2021; Xu et al. 2021; Kojima and Shimizu 2022).

Moderate electric fields (MEF) have also been used in different matrices to extract a variety of compounds. Extractions using MEF range from pectin from passion fruit peel (De Oliveira et al. 2015), inulin from Jerusalem artichoke tuber powder (Gavahian et al. 2018), phenolic compounds from *Pinus pinaster* bark (Ferreira-Santos et al. 2019), anthocyanins from black rice bran (Gavahian et al. 2018), and carotenoids and lipids from microalgae (Jaeschke et al. 2016), among others. Nevertheless, similarly to HHP, studies using MEF to extract lipids from microalgae are limited (De Carvalho Neto et al. 2014; de Souza et al. 2014; Jaeschke et al. 2016).

Based on the above rationale, the aim of the research presented herein was to study HHP (pressure) and MEF (electric field) as technologies to support extraction of EPA-rich fractions from *N. oculata* with the concomitant use of solvents with lower detrimental impact than the ones traditionally used. The main objective was to achieve, at least, similar yields to those obtained using the traditional methodologies.

Both technologies were tested independently and in combination, in order to explore the potential of the technologies per se, and also if their combination could further improve extraction performances. High hydrostatic pressure was used concomitantly with an extraction solvent, while MEF was utilized as a pre-treatment to a subsequent solvent extraction. To the best of our knowledge, this is the first time HHP and MEF are strategically combined to extract lipids from *Nannochloropsis* genus microalgae.

## Materials and methods

### Chemicals

Hexane and EtOH were purchased from Carlo Erba (Vale de Reuil Cedex, Spain), methanol (MeOH) and Chl from Fischer Scientific (Loughborough, UK), diethyl ether (Et_2_O) from Alfa Aesar (Karlsruhe, Germany), and isopropanol (2-PrOH) from VWR (Fontenay-sous-Bois, France).

### Strain and culture conditions

The microalga used in this study was *Nannochloropsis oculata* CCAP 849/1 (SAMS Ltd., Scottish Marine Institute, Scotland, UK), which was grown in 5 L flat-bottomed glass balloons, with artificial seawater medium (ASW) (Darley and Volcani 1969), and an aseptic stream of forced air to ensure aeration and access to light of the entire culture (as bubbling avoided biomass deposition). Growth occurred under continuous light at 65 µmol photons/m^2^/s (LI-1000 DataLogger; LI-COR, USA), provided by cool daylight fluorescent lamps (LUMILUX, L18W/840, OSRAM, Portugal), and at 25 °C, in a climate chamber S600PL (Aralab, Portugal).

The microalga biomass was harvested in late-log/early-stationary phase by centrifugation (1400 g, 4 °C, 5 min). The supernatant was discarded, and the remaining pellet was used for extraction, either directly (wet) or freeze-dried.

### Extraction methodologies

#### Solvent mixtures

Two solvent mixtures (SM) were used: (i) SM_1_—Hex: 2-PrOH (3: 2), as defined by Hara and Radin (1978) and (ii) SM_2_—Et_2_O: EtOH (2: 1) established based on polarity properties to be tested in this study. Biomass was added to the SM and extraction occurred under the conditions described below (specific for each technology used). After extraction, a subsequent purification step was performed through solvent partition (with Na_2_SO_4_ 0.1 mM in a 1: 2 ratio for SM_1_ and 1: 2 water for SM_2_). After mixing, solutions were centrifuged (1400 g for 10 min, at 4 °C) and Hex or Et_2_O (upper layers) were collected to a pre-weighed rotary evaporator glass balloon, followed by solvent evaporation to complete dryness for gravimetric lipid quantification.

For comparative purposes, Folch et al. (1957) extraction (with SM_ref_—Chl: MeOH (2: 1)) was also tested against the two abovementioned solvent mixtures.

#### High hydrostatic pressure (HHP)

High hydrostatic pressure extraction was performed by adding SM to wet (180: 1, v/w) or freeze-dried (90: 1, v/w) biomass, transferring the resulting mixture to polyethylene bags and heat-sealing, avoiding as much as possible the presence of air. The bags were then placed inside the pressure vessel of a high-pressure equipment (Stansted Fluid Power FPG7100, UK) and subjected to the experimental conditions presented in Table 1 (applied at a pressurization rate of 450 MPa/min) using a mixture of water and propylene glycol (60: 40) as the pressurization fluid. Pressure indicated as 0.1 MPa (atmospheric pressure) represents maceration conditions.

**Table 1:** Summary of the HHP and MEF conditions tested using SM_1_

HHP	MEF
MPa/°C/min	(V/cm)/min
0.1/21/15	
0.1/21/30	
200/21/15	0/15
200/21/30	0/30
400/21/15	
400/21/30	3/15
0.1/40/15	3/30
0.1/40/30	
200/40/15	10/15
200/40/30	10/30
400/40/15	
400/40/30	

After pressurization, the bags were opened and SM: biomass mixture was collected; the interior of the bags was washed with 20 mL of Hex or Et_2_O (according to the SM being used), and the pooled SM: biomass mixture was centrifuged; SM fraction was collected and processed according to the procedure previously described for solvent partition.

#### Moderate electric field (MEF)

**Fig. 1:**
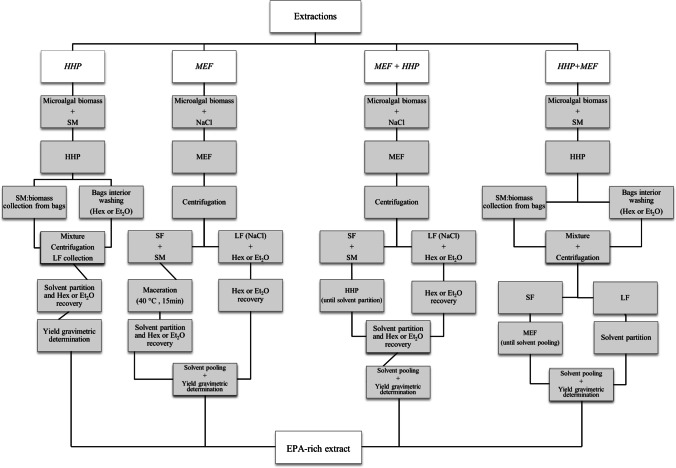
Flow chart of independent and combined extraction methodologies (SF: solid fraction; LF: liquid fraction)

Moderate electric field extraction was conducted in the apparatus described in Pagels et al. (2021). As the system did not allow the use of organic solvents, wet biomass was firstly re-suspended in 0.55 g/L NaCl solution (NaCl: biomass—180: 1, v/w (dry-weight); conductivity of 3 mS/cm) when exposed to the electric field, and only afterwards (after MEF was performed) into the SM. As such, in this case, MEF could be considered a pre-treatment for the subsequent solvent extraction, and not an extraction methodology per se. The steps performed during MEF extraction are presented in Fig. 1. To avoid biomass deposition, gentle agitation by magnetic stirring was applied during MEF.

After exposure to electric field, the NaCl: biomass suspension was centrifuged, the components separated, and biomass was mixed with SM (40 °C, 15 min). Thereafter, SM was separated from the biomass through centrifugation and treated accordingly (partition). The supernatant from NaCl: biomass centrifugation (i.e., NaCl solution) was also extracted (to recover any lipophilic compounds that could have been released from the biomass during MEF) by adding 20 mL of Hex or Et_2_O (according to the SM being used), vortexed for 1 min, followed by centrifugation for phase partition. The solvent was then collected and pooled with the (same) solvent resulting from SM partition, and gravimetric determination of lipids was performed. Table 1 shows the combinations of electric field and time tested. When frequency is indicated as 0 V/cm, biomass was solely exposed to NaCl solution. Due to restrictions in operating temperature for certain conditions related with the MEF apparatus, all extractions were performed at 40 °C.

#### Combined extraction (MEF + HHP and HHP + MEF)

A combination of technologies was performed in sequential mode and using the two possible sequences of processes (MEF followed by HHP and HHP followed by MEF). MEF + HHP was performed by collecting the NaCl: biomass solution resulting from the last step of MEF per se (Fig. 1), centrifuging the mixture and collecting biomass to perform HHP extraction. After partitions of SM_2_ resultant from both extraction procedures, the organic solvent containing the lipids was pooled for gravimetric determination of the lipids content. A similar procedure was used for HHP + MEF.

Table 2 presents the different conditions of parameters (electric field and/or pressure) applied in HHP and MEF combined assays. Individual HHP and MEF extractions were also performed to serve as controls. In order to allow comparisons, all extractions and/or exposures were conducted at 40 °C for 15 min, except for HHP, which was performed at 21 °C, according to the conclusions reached in HHP independent extractions.

**Table 2:** Conditions used in combinations of HHP with MEF, performed with experimental conditions selected as the most promising, according to yields obtained in individual extractions (Table 1)

Conditions	
MEF (3 V/cm)	
NaCl^*^	
HHP (200 MPa)	
MEF (3 V/cm) + HHP (200 MPa)	
HHP (200 MPa) + NaCl*	
HHP (200 MPa) + MEF (3 V/cm)	

^*^Exposure of biomass to NaCl solution

#### Fatty acid profile

Fatty acid profile was determined by gas chromatography with flame ionization detector (GC-FID) according to the method described by Sousa et al. (2022), with slight modifications: following solvent evaporation (Hex or Et_2_O, according to the SM used for extraction) and gravimetric assessment of lipid content, resulting lipids were resuspended in the same solvent, transferred to borosilicate glass tubes and evaporated with a gentle stream of nitrogen. Thereafter, the remaining procedure and the conditions utilized in gas chromatography analyses of the collected FAME were performed according to the protocols described in Sousa et al. (2022).

### Statistical analysis

SPSS 20 software (SPSS, Chicago, IL, USA) was used to perform statistical analyses. Samples were compared through analysis of variance (ANOVA) with Tukey as post hoc when all required assumptions were met. Otherwise, ANOVA was substituted by Kruskal–Wallis, and pair-wise comparisons were performed using Mann–Whitney. A significance level of 0.05 was used in all analyses. All experiments were performed in triplicate, and all results are expressed on a dry-weight basis.

## Results

Lipid extraction procedures referred in literature usually use organic compounds such as Chl (not allowed in the food industry), which are hazardous for human health and the environment and, as such, there is an increasing need to find adequate substitutes (Wan Mahmood et al. 2017). Besides exploring different solvents and solvent mixtures, another approach consists in using technologies to enhance extraction yields of less hazardous solvents that may otherwise not be as efficient as the ones employed in the traditional methodologies. Those technologies aimed at increasing solvent penetration by enhancing cellular membrane permeability or even whole cell destruction.

**Fig. 2:**
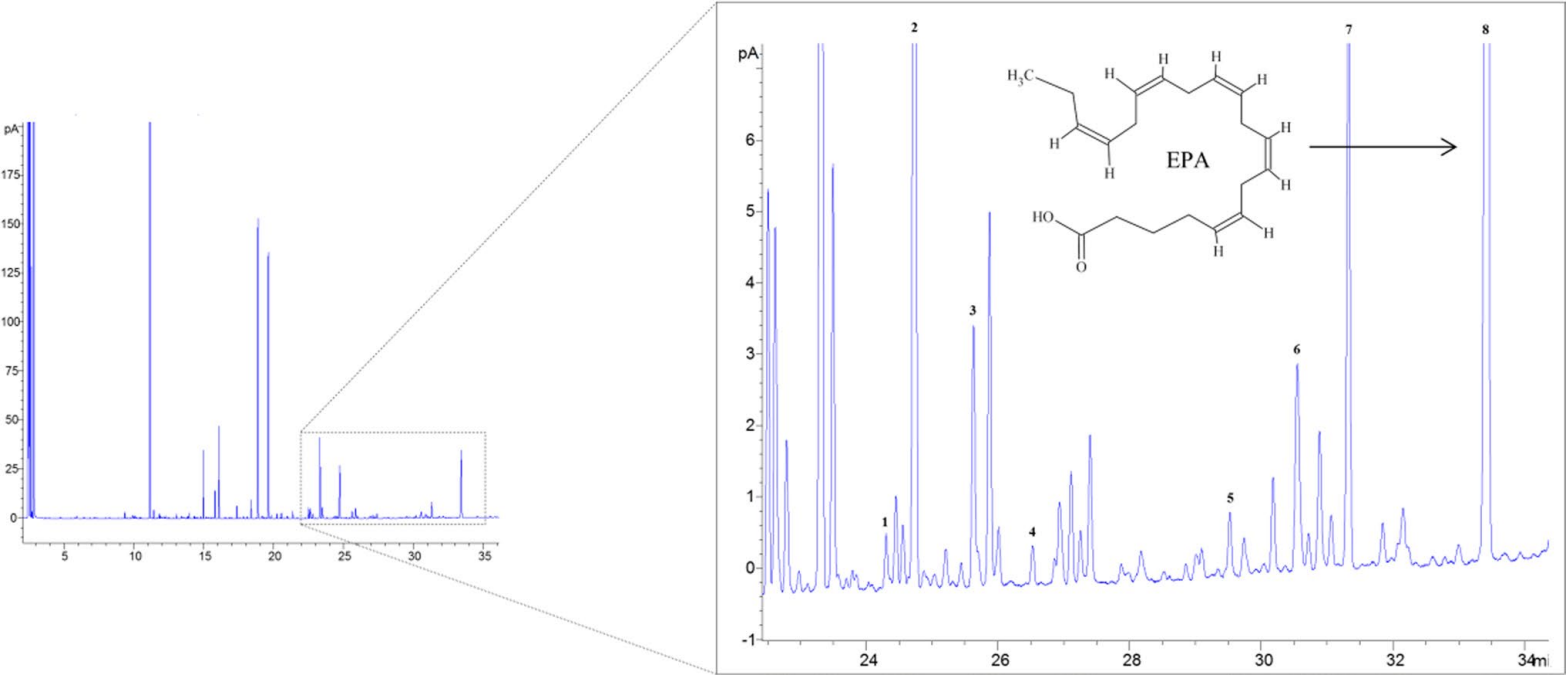
GC-FID chromatogram with the identification of the polyunsaturated fatty acids (PUFA) found in the extracts (1—C18:2 c9t12; 2—C18:2 c9c12; 3—C18:3 c6c9c12; 4—C18:3 c9c12c15; 5—C20:2 c11c14; 6—C20:3 ω6; 7—C20:4 ω6; 8—C20:5 ω3 (EPA))

In the present research work, HHP and MEF were tested independently and in combination, with the goal of increasing the efficiency of different extraction mixtures (using solvents with lower detrimental impacts than the ones traditionally employed), to obtain lipid extracts rich in EPA (Fig. 2) from *N*. *oculata*.

Extractions were assessed by measurement of lipid and fatty acids contents, the latter either in terms of EPA per lipid content (reflecting specificity of extraction in terms of compounds selectivity) or per biomass (total amount of EPA recovered). All experiments, independent of the technology used, were performed at a maximum of 40 °C to prevent thermal degradation of unsaturated fatty acids, as temperatures of 50 °C and above have been reported to significantly degrade EPA (Hǎdǎruga et al. 2016).

### Extraction methodologies

Initially, HHP and MEF extractions were performed using Hex: 2-PrOH (SM_1_) as SM, which is the solvent mixture presented by Hara and Radin (1978) as a less hazardous alternative to the classical chloroform-based solvent mixtures described by Bligh and Dyer (1959) and Folch et al. (1957). However, the results obtained in the first HHP and MEF extractions, which will be presented further on, did not show relevant improvements in EPA extraction yields, probably due to the solvents comprised in SM_1_. Furthermore, it has been reported that solvents (or solvent mixtures) with higher polarities than the ones composing SM_1_ may produce better extraction yields concerning both lipids and PUFA, namely, EPA (Pieber et al. 2012; Balasubramanian et al. 2013).

Considering the unsatisfactory lack of improvements in yields obtained in HHP and MEF extractions (and the possible phenomenon behind the HHP results discussed below) and the fact that the traditional methodology used for lipid extraction uses Chl: MeOH, which are solvents more polar than the ones used in SM_1_, it was decided to test a new extraction mixture, with a higher average polarity index (PI) than SM_1_.

#### Comparative evaluation of solvent mixtures

A mixture of Et_2_O: EtOH (SM_2_) was chosen as it has a PI of 3.6, which is an intermediate value between SM_1_ previously used (PI 1.6), and SM_ref_ used in Folch’s method (PI 4.4) (Snyder 1978). In order to evaluate the extraction efficacy of SM_2_, independent extractions (without HHP or MEF) using freeze-dried biomass were performed at 21 °C for 15 min, with 3 solvent mixtures: Hex: 2-PrOH (SM_1_, previously used; PI 1.6), Et_2_O: EtOH (SM_2_; PI 3.6), and Chl: MeOH (SM_ref_; PI 4.4), for reference comparison purposes.

**Fig. 3:**
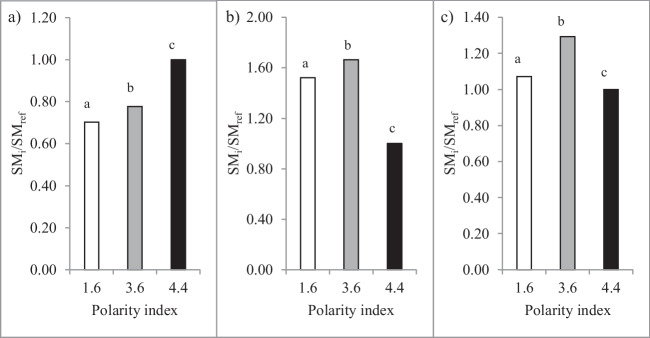
Normalized ratios (SM_i_/SM_ref_) of lipid (mg_lipid_/g_biomass_; **a**) and EPA (mg_EPA_/g_lipid_ and mg_EPA_/g_biomass_; **b**, **c**, respectively) yields with different solvent mixtures, per lipid (**b**) and per biomass (**a** and **c**). PI 1.6 (SM_1_), PI 3.6 (SM_2_), and PI 4.4 (SM_ref_)

Concerning total lipids, results (Fig. 3a) showed that SM_2_ extracted more lipids than SM_1_, although both were less effective than SM_ref_. Regarding EPA per lipid content (Fig. 3b), SM_2_ was the SM that presented the best result, 1.7-fold increase of the yield obtained with SM_ref_, followed by SM_1_, which presented a 1.5-fold increase over the SM_ref_ EPA yield. Concerning EPA per biomass (Fig. 3c), SM_2_ extracted the highest content (3.0 mg_EPA_/g_biomass_) which, in comparison with the obtained using SM_ref_ corresponded to a 1.3-fold increase, followed by SM_1_ (1.1-fold increase over SM_ref_ yield).

#### High hydrostatic pressure (HHP)

##### SM_1_ (Hxn: 2-PrOH)

In order to assess HHP extraction, freeze-dried biomass was initially used, since there are evidences that biomass water content negatively affects lipid extraction efficiency (Chen et al. 2011; Balasubramanian et al. 2013; Islam et al. 2014). As previously mentioned, the first HHP extraction experiment was performed using SM_1_, and pressure (0.1, 200, and 400 MPa), temperature (21 and 40 °C), and time (15 and 30 min) were tested in the combinations described in Table 1; extraction at 0.1 MPa, 21 °C, and 15 min was used as the control.

**Fig. 4:**
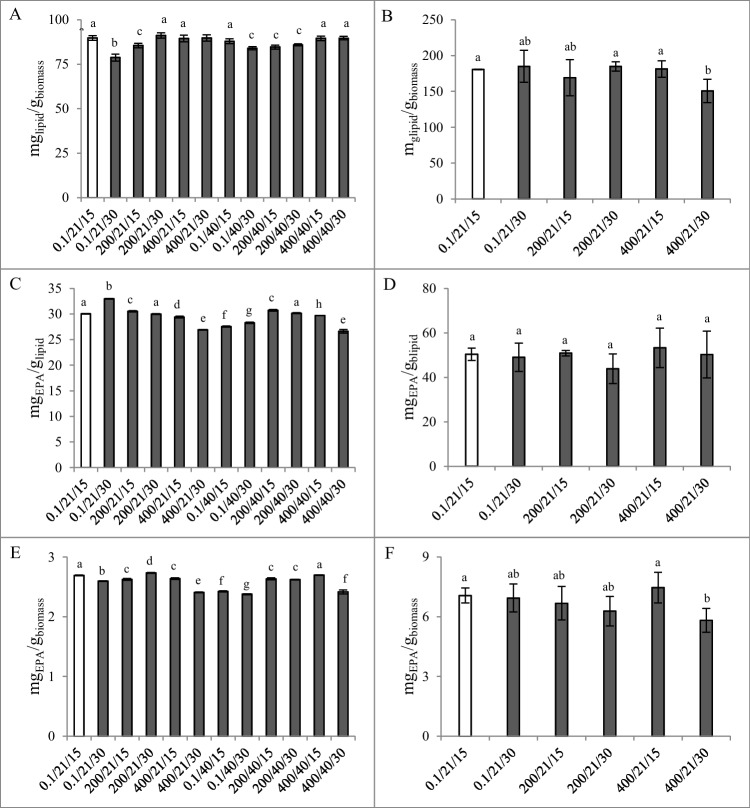
Lipid (**A**, **B**), EPA per lipid (**C, D**), and per biomass (**E, F**) yields of control and HHP extractions at different pressure, temperature, and time, presented as pressure (MPa)/temperature (°C)/time (min), performed with SM_1_ (**A**, **C**, and **E**) and SM_2_ (**B, D**, and **F**). Different lowercase letters indicate significant differences (*p* < 0.05) between yield values

Lipid extraction results (Fig. 4A) showed that, when compared with the control, none of the tested conditions significantly (*p* > 0.05) improved lipid extraction yield. Despite some statistically significant differences (*p* < 0.05) between the distinct conditions assessed, there was no clear trend regarding any of the technological parameters assessed.

Regarding EPA content (Fig. 4C and D), the overall behavior was similar to the observed concerning total lipids. Pertaining to mg_EPA_/g_lipid_ (Fig. 4C), only three extraction conditions resulted in significantly (*p* < 0.05) higher yields than the control (0.1/21/15, which yielded 30.0 mg_EPA_/g_lipid_), which were 0.1/21/30, 200/21/15, and 200/40/15 yielding 32.9, 30.5, and 30.7 mg_EPA_/g_lipid_, respectively. As abovementioned, no clear trend could be observed in terms of any of the tested parameters consistently impacting extraction, which was also the case regarding the amounts of EPA per biomass. As far as mg_EPA_/g_biomass_ (Fig. 4E) is concerned, the EPA extraction yield obtained in the control conditions (2.69 mg_EPA_/g_biomass_) was only surpassed by applying HHP at 200 MPa, at the same temperature and for 30 min, which yielded a very slightly higher 2.73 mg_EPA_/g_biomass_ (*p* < 0.05).

In this sense, considering all the above results, there was no positive impact of applying HHP (pressure) in the extraction process, on both lipids and EPA extraction yields.

##### SM_2_ (Et_2_O: EtOH)

The following step was to perform HHP independent extraction with SM_2_, although only at 21 °C, to avoid the combination of the vessel temperature with the adiabatic compression heating which could degrade the extracted lipids and EPA.

Similar to the observed in the first HHP extractions, results (Fig. 4B, D and F) showed that, in comparison with the extraction performed in the control conditions (0.1/21/15), HHP was not able to significantly increase (*p* > 0.05) lipid and EPA extraction yields. Based on these results, the pressure of 200 MPa, at 21 °C for 15 min was chosen as the HHP condition to be utilized in the experiments in which HHP and MEF were to be combined, given the lower energy costs associated.

#### Moderate electric fields (MEF)

##### SM_1_ (Hxn: 2-PrOH)

Concerning MEF extraction, and as previously mentioned, initial extractions were performed using SM_1_. Considering that the biomass had to be suspended in a NaCl solution, wet biomass was used, which also enhances industrial applicability.

Electric field (3 and 10 V/cm) and time (15 and 30 min) were the technological parameters tested (combinations presented in Table 1). Extractions without biomass exposure to an electric field (0 V/cm) were performed as controls, in which biomass was nonetheless in contact with the NaCl solution, for 15 or 30 min. All extractions were performed at 40 °C due to limitations/restrictions of the MEF equipment concerning refrigeration when applying an electric field of 10 V/cm.

**Fig. 5:**
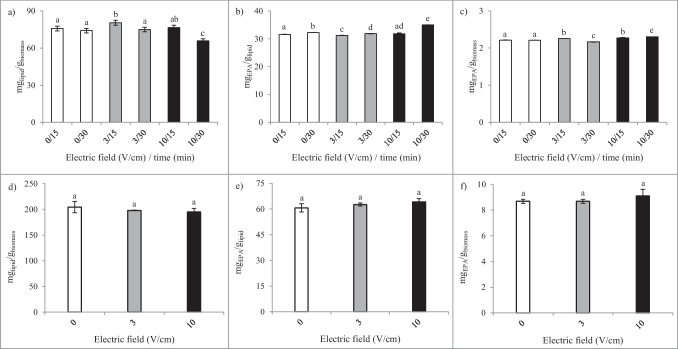
Lipid (**a**, **d**), EPA per lipid (**b**, **e**) and per biomass (**c**, **f**), yields of control, and MEF extractions at different conditions, performed with SM_1_ (**a**–**c**) and SM_2_ (**d**–**f**). Different lowercase letters indicate significant differences (*p* < 0.05) between yield values

Lipid extraction yields (Fig. 5a) obtained with MEF showed, when compared with control (0/40/15), an increase in lipid yield (*p* < 0.05) only when an electric field of 3 V/cm was applied for 15 min, for which the highest amount of lipids (80.4 mg_lipid_/g_biomass_) was obtained, representing a 6% increase comparatively with the control. In the remaining conditions tested and despite the application of electric field, except for the 10/40/30 extraction condition which yielded lower lipid content, no significant differences (*p* > 0.05) were obtained between these extractions and the ones in which biomass was only exposed to NaCl solution.

The only parameter that seemed to affect extraction, although to a low extent, was time; indeed, prolonged extraction time (30 min vs. 15 min) yielded significantly lower amounts of lipids (*p* < 0.05); specifically, there was a decrease from 80.4 to 75.0 mg_lipid_/g_biomass_ at 3 V/cm and from 76.5 to 65.9 mg_lipid_/g_biomass_ at 10 V/cm. In this sense, the highest lipid yield was therefore obtained when extraction was performed at 3 V/cm for 15 min, while the lowest was registered at 10 V/cm, for 30 min. Such results appeared to indicate that there might be a threshold above which the electric field, in combination with the time during which it is applied, could degrade the lipids.

Concerning the amounts of EPA per fat content (Fig. 5b), the highest value was obtained in MEF at 10 V/cm for 30 min (35.0 mg_EPA_/g_lipid_), followed by 0 V/cm also for 30 min (32.2 mg_EPA_/g_lipid_). In this sense, time impacted extraction yields in an opposite fashion to the previously observed, since increasing extraction time concomitantly increased the EPA amounts. Increasing extraction time from 15 to 30 min resulted in higher yields, in all conditions assessed, which means that for each electric field applied (or no electric field), prolonged exposure of the biomass/SM mixture increased extraction efficiency, yielding significantly (*p* < 0.05) higher EPA contents. Concerning a possible correlation between the application (or not) of electric field and the EPA yields, in terms of mg_EPA_/g_lipid_, results showed that, despite being significant (*p* < 0.05), the differences between extractions were not relevant, except for the extraction performed at 10 V/cm for 30 min.

Pertaining to EPA per biomass (Fig. 5c), the highest amount was also obtained at 10 V/cm for 30 min (2.30 mg_EPA_/g_biomass_). However, the difference between this extraction and the remaining was lower than the previously observed regarding mg_EPA_/g_lipid_. In this case, and in line with the previous findings, no coherent behavior was observed between the amounts of EPA extracted and the alterations in the different parameters assessed (electric field and time). The overall absence of relevant differences between the extractions indicated that the electric fields applied when MEF was performed did not significantly impact *N. oculata* cells to the desired extent (increase extraction yield, more specifically, mg_EPA_/g_biomass_).

##### SM_2_ (Et_2_O: EtOH)

Similar to HHP, MEF independent extraction was also assessed utilizing SM_2_. In this case, to try to understand if the results previously obtained concerning the (absence of) impact of the electric field on lipid and EPA extraction yields was maintained, extractions were performed with electric fields of 0, 3, and 10 V/cm and were only performed for 15 min, which was the time frame in which the best results concerning mg_EPA_/g_biomass_ were obtained.

The lipid and EPA yields obtained when applying the distinct extraction conditions were not significantly different (*p* < 0.05) (Fig. 5d–f). These results showed, as in the previous independent MEF experiment, that the electric field did not impact in any way the extraction efficiency of the SM. The absence of such differences in the extractions indicated that, seemingly, the applied MEF did not produce an impact on *N*. *oculata* cells.

Considering these findings and to further corroborate that the yields obtained in MEF were not originated by applying the electric field, when assessing the combinations of the extraction technologies, besides independent MEF (which was performed at 3 V/cm), NaCl exposure (0 V/cm) was also tested.

### Combined extractions (MEF + HHP and HHP + MEF)

Having tested HHP and MEF independently, combinations of both were also tested to try improving yields. In these experiments, SM_2_ was used, and microalgae biomass was always in wet form, for comparative analysis. Nonetheless, besides the combinations, MEF (and NaCl exposure) and HHP were again tested independently, as control assays.

**Fig. 6:**
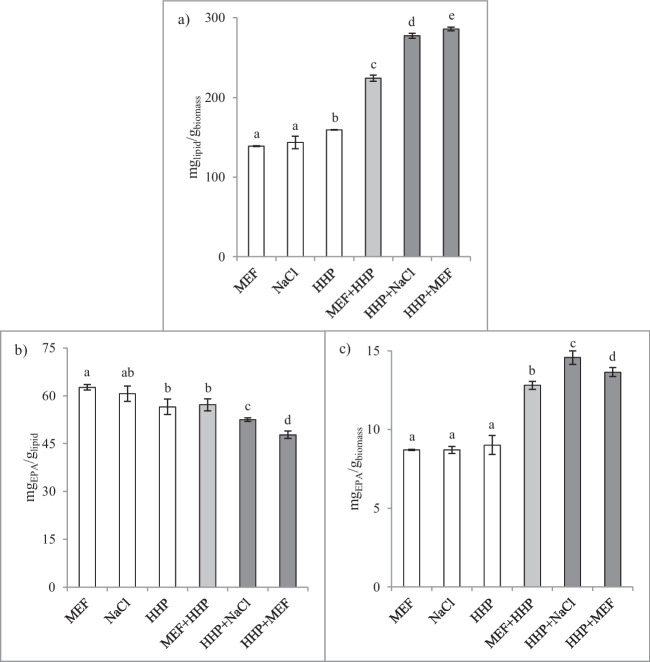
Lipid (**a**) and EPA per lipid (**b**) and per biomass (**c**) yields obtained with different extraction technologies and combinations thereof. Different lowercase letters indicate significant differences (*p* < 0.05) between yield values

Results pertaining to the independent extractions showed that HHP yielded a slightly higher (*p* < 0.05) lipid content than independent MEF and NaCl extractions (Fig. 6a).

Regarding the combinations of technologies, results showed positive outcomes, with significant (*p* < 0.05) increases in lipid yield (Fig. 6a) being obtained. Concerning MEF + HHP, pre-treatment of biomass by MEF increased extraction efficiency of HHP, since in comparison with HHP independent extraction, lipid yield in MEF + HHP was higher (159 vs. 224 mg_lipid_/g_biomass_, respectively, representing a 41% increase). Moreover, it was observed that MEF + HHP presented a higher yield than MEF alone (224 vs. 139 mg_lipid_/g_biomass_, respectively, a 61% increase), indicating that the pressure exerted in HHP increased the extraction of biomass previously exposed to MEF. Since in MEF independent extraction, as part of the methodology, a solvent extraction was performed after exposure of the biomass to MEF, this result showed that the conditions utilized in MEF rendered the cells more susceptible to be affected by the pressure applied in the subsequent HHP treatment. This phenomenon was responsible for the increased extraction yields observed in MEF + HHP, since the lipid and EPA amounts obtained were, as abovementioned, higher than those obtained in MEF or HHP independent extractions.

The combinations of HHP + MEF and HHP + NaCl were able to yield even higher lipid contents (286 and 277 mg_lipid_/g_biomass_, respectively). In these cases, the increases, in comparison with the independent extractions, must have resulted from the fact that in the methodologies two extractions were performed, namely, one during HHP and the other after exposure to MEF or NaCl conditions.

Pertaining to fatty acids per lipid (Fig. 6b), a contrasting behavior was observed, since independent extractions (and MEF + HHP) obtained the highest yields. The highest EPA yields were registered in MEF, followed by NaCl (62.6 and 60.6 mg_EPA_/g_lipid_, respectively). The lowest EPA contents were yielded when two extractions were performed, namely, HHP + NaCl and HHP + MEF (52.6 and 47.7 mg_EPA_/g_lipid_, respectively). These results indicate that performing two extractions must have increased the extraction of lipophilic compounds other than EPA, which resulted in lower EPA contents per lipid content. Nonetheless, overall extraction of EPA was increased since extraction yield (mg_lipid_/g_biomass_) was also increased.

Regarding EPA per biomass (Fig. 6c), results showed that the amounts extracted presented a behavior similar to the one observed concerning lipid yields. Combinations of technologies yielded higher amounts of EPA than independent extractions, although in this case, the combination HHP + NaCl was the extraction that yielded the highest, followed by HHP + MEF (14.57 and 13.64 mg_EPA_/g_biomass_, respectively).

## Discussion

### Extraction methodologies

#### Comparative evaluation of solvent mixtures

Regarding the comparison between solvent mixtures, SM_ref_ was able to extract the highest lipid content, followed by SM_2_ and SM_1_ in descending order of percentage yield. This result is in agreement with the findings of Teo and Idris’ (2014) study, which compared Folch and Hara and Radin methods to extract lipids from *Nannochloropsis* sp. and found the Folch method to register higher yield than the Hara and Radin counterpart (c.a. 8 and 5.25% lipid yields, respectively). A plausible explanation for the fact that SM_ref_ yielded the highest lipid content, although the EPA content per lipid was the lowest, may be due to the extraction of other types of lipophilic compounds such as carotenoids or other pigments.

Results showed that SM_2_ yielded higher lipid content than SM_1_ (90.5 vs. 81.8 mg_lipid_/g_biomass_) and was able to slightly increase extraction of EPA per lipid, which resulted in higher content per biomass. Also (as abovementioned), comparatively to SM_ref_, EPA per biomass yield was considerably higher. Concerning the detrimental (health and environmental) impacts of the solvents, particularly the “main” solvents of the SM, these have been targeted over the last years in several publications and manuals (Prat et al. 2015; Alder et al. 2016; Sneddon 2017; Calvo-Flores et al. 2018; Joshi and Adhikari 2019). In terms of environmental impact, Et_2_O may be classified as an environmentally favorable solvent, due to its low environmental impact of production and relatively high credits for energy recovery (Capello et al. 2007), as well as not being considered to bioaccumulate, hence not being persistent, and to display very low toxicity to aquatic life (GPS Safety Summary 2015).

Pertaining to human health, Et_2_O is considered to present the lowest risk and, generally, both Et_2_O and Hxn have been considered less harmful than Chl, which has been classified as a carcinogen (Prat et al. 2015; Alder et al. 2016; Calvo-Flores et al. 2018). However, due to its toxicity Hxn has been, in some cases, considered to be as harmful to human health as Chl (Prat et al. 2015) and both are classified in the United States as hazardous airborne pollutants (Joshi and Adhikari 2019).

Owing to those characteristics of the solvents, extractions with lower-toxicity alternatives have been explored, such as in the study of Jiménez Callejón et al. (2020), in which a two-step extraction procedure (with hexane, followed by ethanol) was utilized to obtain EPA from *Nannochloropsis* sp.. The process was able to extract 74.7% of the overall EPA content of the microalga.

Considering all of the above, it was decided that SM_2_ was a viable alternative to be used in the second round of the independent HHP and MEF extractions and in the combination experiment in which both technologies were sequentially utilized.

#### High hydrostatic pressure (HHP)

##### SM_1_ (Hxn: 2-PrOH)

High hydrostatic pressure extraction is achieved through a large differential pressure between the outside and inside of the cells, which can result in a deformation, or even disruption, of the cellular membranes, thus originating a rapid permeation of the solvent, increasing extraction efficiency. Solubility of several compounds can also be increased as consequence of HHP, which will also increase extraction yields (Khan et al. 2018; Monteiro et al. 2018). However, the results obtained herein, when HHP was applied to extract lipids and EPA, are not in accordance with such principles as exposure of the biomass/SM binomial to HHP did not increase extraction yields.

Compression heating is a phenomenon which results from the work of compression against intermolecular forces and leads to a temperature increase of compressible materials. When heat does not enter or leave the system, it is designated adiabatic compression heating. This phenomenon occurs in HHP and can result in a degradation of thermo-sensitive compounds (Sun et al. 2018). Khan et al. (2018) stated that during HHP process, for every 100 MPa increase in pressure, a 3 °C rise in temperature occurred. The research work by Sun et al. (2018), studying six different organic solvents, showed that polarity had a negative correlation with compression heating of liquid materials. Lipid yield has been shown to decrease with temperature increase above a determined threshold, as in Ali and Watson’s (2016) study, in which temperature increase above 42.2 °C resulted in lower lipid yields.

Regarding the effect of the pressure, a study performed by Kojima and Shimizu (2022) revealed a positive impact of hydrostatic pressure in lipid yield of *Chlorella vulgaris*, using an ionic liquid ([1-ethyl-3-methylimidazolium acetate]-DMSO), at 25 °C for 60 min. However, such improvement was only observed at 50 MPa, since a decrease in lipid yield was observed with increasing pressure thereafter. Bueno et al. (2020), using EtOH and limonene to extract PUFA from *Porphyridium cruentum* and *Haematococcus pluvialis*, found EPA yields similar to those obtained in control extraction when pressures of 100 and 300 MPa were applied, at 50 °C for 20 min. However, when HHP was performed at a higher pressure of 600 MPa both PUFA and EPA yields decreased. These results are in accordance with the findings presented herein. However, a study performed by the same research group (Gallego et al. 2021) applying those methodology and technology on *Nannochloropsis oceanica* found that the increase of pressure from 100 up to 600 MPa did not significantly impact PUFA and EPA yields and that only 100 MPa were required to obtain yields similar to those of the conventional extraction. Contrastingly, Xu et al. (2021) applied HHP to microalgae slurries, using Chl: MeOH (SM_ref_) as SM and without temperature control, and reported increased lipid yields at 400 and 600 MPa, when compared with control and extractions performed at lower-pressure magnitudes.

Pressure used in high hydrostatic pressure processing has also been associated with lower lipid yields at low temperatures, as higher lipid oxidation values were registered in turbot (*Scophthalmus maximus*) muscle (Chevalier et al. 2001) and carp (*Cyprinus carpio*) fillets (Sequeira-Munoz et al. 2006) by pressure treatments of 180 MPa at 4 °C. Thus, adiabatic compression heating may have compromised the results obtained with HHP, especially at 40 °C, since the compressions may have led to temperature increases that lead to degradation of the unsaturated fatty acids, namely, EPA (Hǎdǎruga et al. 2016). In this sense, increased amounts of lipids and PUFA (and among them, EPA) which could potentially have been extracted by the increased pressures may have also been degraded due to the higher temperature resultant from adiabatic compression heating.

Overall, these results revealed that HHP did not improve extraction yields of lipids and EPA, when using freeze-dried biomass and SM_1_. Therefore, a second round of experiments was performed using the improved SM of higher polarity (SM_2_). Additionally, considering potential industrial application, wet biomass was used.

#### Moderate electric fields (MEF)

##### SM_1_ (Hxn: 2-PrOH)

The fact that different behaviors were obtained regarding the impact of MEF on the extraction yields of lipids (mg_lipid_/g_biomass_) and those of EPA (mg_EPA_/g_lipid_ and mg_EPA_/g_biomass_) indicated that when different extraction conditions were applied, distinct lipophilic compounds were extracted.

As aforementioned, extraction using 10 V/cm for 30 min yielded the highest EPA contents (mg_EPA_/g_lipid_ and mg_EPA_/g_biomass_), despite being the one which yielded the lowest lipid content (mg_lipid_/g_biomass_), meaning that the lipid fraction obtained in this extraction condition presented the highest EPA percentage (3.5% of total lipids). However, when MEF was applied at 3 V/cm for 15 min, the highest lipid yield was registered, although in terms of EPA per biomass, it was only the second highest (2.8% of total lipids). As previously mentioned, such results indicate that when the electric field applied was lower, the lipid fraction extracted was composed of a higher percentage of lipophilic compounds distinct from EPA.

Extraction using MEF is based on the principle that a transmembrane potential occurs when a cell is exposed to an electric field; when it reaches a certain threshold, it promotes electro-permeabilization of the membrane. This phenomenon is also designated as electroporation, as the electric field leads to the creation of pores in the cellular membrane, or to the enlargement of existing ones. Depending on the intensity of the electric field, electroporation can be temporary (reversible) or permanent (irreversible) (Joannes et al. 2015; Geada et al. 2018). Electroporation can increase the compound flux through the membrane, or it can lead to cell damage and/or destruction. Exposure to an electric field, and the consequent electroporation (when taking place), can result in spontaneous release of certain compounds (Postma et al. 2016; Geada et al. 2018), case in which the application of moderate electric fields can be considered an extraction methodology, or it can be used as a pre-treatment to facilitate subsequent extraction (Eing et al. 2013).

Electrotechnologies such as PEF and MEF have been used for extraction of valuable compounds from microalgae. Although PEF is most studied, some studies have explored MEF which, because of using lower electric fields, can present a better cost/efficiency ratio and can also have a lower destructive impact on cells. Such moderation can be a positive aspect due to the lower amount of other intracellular compounds released, which decreases the number and cost of the subsequent purification steps.

The positive effects of electrotechnologies in lipid extraction have been described by few authors. Jaeschke et al. (2016) studied the effect of MEF on the extraction of lipids and carotenoids from freeze-dried *Heterochlorella luteoviridis*, using ethanol and voltages from 0 to 180 V (at 60 Hz). MEF was used as pre-treatment, since after MEF, a subsequent diffusive step was performed. Results showed that with 90 V and 75% EtOH, they could obtain lipid yields correspondent to 83% of the lipids obtained with the Bligh and Dyer method (using Chl: MeOH, 1: 2). de Souza et al. (2014) and De Carvalho Neto et al. (2014) also used MEF to extract lipids; the authors used electroflotation by alternating current (EFAC), in which EFAC was used as a pre-treatment, being the biomass subsequently freeze-dried and extracted according to the Bligh and Dyer method. The studies were performed at 12 V, and several frequency ranges were tested, from 0–1.56 kHz to 0–50 kHz. In De Carvalho Neto et al.’s (2014) study, time was also evaluated with treatments at 0–1.56 kHz from 40 to 140 min. Results showed that frequency ranges did not influence lipid yields and that lipid yield increased by 9 to 14% with treatments of 40 and 140 min, respectively. Concerning de Souza et al.’s (2014) work, the highest lipid yield achieved with EFAC was 24.8% ± 7.1%, while control conditions without pre-treatment yielded 4.8%.

The results obtained herein can be compared with those obtained by de Souza et al. (2014) and De Carvalho Neto et al. (2014), since MEF was also used as a pre-treatment prior to extraction. In the present work, the extraction efficiency was much lower (less than a third) than the 24% found in de Souza et al. (2014), since only 8% lipids were achieved, and less than half of the 14% increase observed by De Carvalho Neto et al. (2014) as herein an increase of 6% in lipid yield was obtained for the treatment with the highest lipid extraction (3 V/cm for 15 min). The difference between yields may have resulted from intrinsic differences in biomass, as in those studies, biomass was composed of a mixture of microalgae, some of which could have membranes easier to permeate. Moreover, the extraction SM utilized in both studies as control for comparison purposes (Bligh and Dyer 1959) was different from those tested herein (SM_1_ and SM_2_).

The fact that MEF did not exert a more significant effect in extraction yields could indicate that MEF treatment only produced temporary electroporation, which allowed the release of some lipids during the time period in which cellular membrane was more permeable. Afterwards, when the stimulus stopped, the membrane recovered and extraction was similar to the extraction of biomass when not subjected to MEF. The abovementioned hypothesis is sustained by the observation that ca. 10% of the lipids separated in MEF extractions were coming from the supernatant obtained after centrifugation of the solution which had been exposed to MEF (microalgal biomass in NaCl solution). The remaining lipids were obtained in the next step of the extraction process, in which biomass was used for extraction using simple contact with SM. As such, these results were indicative of release of lipids during the first stage of the process.

On the other hand, concerning exposure to NaCl, given that the salt concentration in the growth medium used for *N*. *oculata* (ASW) was 23.6 g/L and that in the solution used in MEF was 0.55 g/L, this makes the solution used in MEF a hypotonic solution. As such, one could hypothesize that contact of the cells with such solution could have resulted in swelling of the cells (turgidity), consequently leading to eventual cell disruption, or at least cause sufficient turgidity to stress the cells enough to destabilize them to facilitate extraction. Such release could explain the results obtained when performing MEF at 10 V/cm for 30 min. The electric field may have been high enough to, with the prolonged exposure, cause degradation of the lipids that were released. In that case, if the lipids degraded were other than EPA, it would result in a lower lipid yield and a higher EPA percentage within the lipid extract, which was the case. Moreover, the lipid yield registered in those conditions (10 V/cm for 30 min) was ca. 10% lower than the remaining yields, which would be in accordance with degradation of the lipids released throughout the period in which biomass was exposed to the electric field.

Nonetheless, the similarity between the values with (except 10 V/cm for 30 min) and without electric field could indicate that the yields had been the result of cell lysis (rupture of the cells) due to osmotic shock, and not of electroporation.

The osmotic principle has been used to for extraction purposes, in a methodology designated “osmotic shock,” which can be used to rupture cellular membranes and extract compounds. The principle is that in the sudden presence of a solution with a different salt concentration than the intracellular, there is an imbalance of the osmotic pressure between the inside and outside of the cells. In the case that the solution is hypotonic (salt concentration is lower than intracellular), diffusion will occur in the opposite direction, meaning that water will enter the cell to equilibrate osmotic pressure, resulting in a swelling of the cells, which can eventually lead to a rupture/burst of cellular membrane (turgidity). In both situations, the rupture of cellular membrane will result in the release of intracellular components, among which are lipids (Halim et al. 2021).

### Combined extractions (MEF + HHP and HHP + MEF)

As previously mentioned, to the best of our knowledge, this is the first work in which the two technologies are strategically combined to increase the yield of *Nannochloropsis* lipid extraction. Overall, the results regarding the independent extractions are in accordance with the findings of the previous experiments and, concerning the combinations in which two extractions were performed, the observed increased yields were to be expected, since biomass was exposed twice to SM. Combinations of HHP and MEF technologies were able to increase both lipids and EPA yields, enabling higher amounts per biomass to be obtained than when each technology was applied independently.

Among the different combinations tested, the HHP + NaCl combination presented the highest values. Eventually, exposure of biomass to MEF extraction conditions caused the cells to be prone to be impacted by pressure applied in HHP, resulting in higher EPA yields. Conjugating HHP extraction with subsequent extraction by MEF (or NaCl exposure) further increased extraction yields, although, as abovementioned, such increases are resultant from the fact that biomass is extracted twice since two extractions are performed when HHP and MEF (or NaCl exposure) are combined.

The conditions applied in the combinations of the technologies and, particularly, the SM_2_ utilized, presented higher EPA yields than those obtained using the traditional methodologies (conditions and solvent mixtures) and, as such, the objective of finding alternative methodologies to extract EPA from *N*. *oculata* was met.

Considering industrial application of such extraction methodologies (and combinations thereof), despite the increased yields and lower environmental impact provided by the innovative approach, in comparison with the “classical” extraction methodologies and solvents, an economical assessment of the process needs to be further performed (cost–benefit analysis).

## Data Availability

The datasets generated during and/or analyzed during the current study are available from the corresponding author on reasonable request.

## References

[CR1] Alder CM, Hayler JD, Henderson RK, Redman AM, Shukla L, Shuster LE, Sneddon HF (2016). Updating and further expanding GSK’s solvent sustainability guide. Green Chem.

[CR2] Ali M, Watson IA (2016). Microwave thermolysis and lipid recovery from dried microalgae powder for biodiesel production. Energy Technol.

[CR3] Balasubramanian RK, Yen Doan TT, Obbard JP (2013). Factors affecting cellular lipid extraction from marine microalgae. Chem Eng J.

[CR4] Baumgardt FJL, Filho AZ, Brandalize MV, Da Costa DC, AntoniosiFilho NR, Abreu PCOV, Corazza ML, Ramos LP (2016). Lipid content and fatty acid profile of *Nannochloropsis*
*oculata* before and after extraction with conventional solvents and/or compressed fluids. J Supercrit Fluids.

[CR5] Bligh E, Dyer W (1959). A rapid method of total lipid extraction and purification. Can J Biochem Physiol.

[CR6] Briones-Labarca V, Giovagnoli-Vicuña C, Cañas-Sarazúa R (2019). Optimization of extraction yield, flavonoids and lycopene from tomato pulp by high hydrostatic pressure-assisted extraction. Food Chem.

[CR7] Bueno M, Gallego R, Chourio AM, Ibáñez E, Herrero M, Saldaña MDA (2020). Green ultra-high pressure extraction of bioactive compounds from *Haematococcus*
*pluvialis* and *Porphyridium*
*cruentum* microalgae. Innov Food Sci Emerg Technol.

[CR8] Calvo-Flores FG, Monteagudo-Arrebola MJ, Dobado JA, Isac-García J (2018). Green and Bio-based solvents. Top Curr Chem.

[CR9] Capello C, Fischer U, Hungerbühler K (2007). What is a green solvent? A comprehensive framework for the environmental assessment of solvents. Green Chem.

[CR10] Chen M, Chen X, Liu T, Zhang W (2011). Subcritical ethanol extraction of lipid from wet microalgae paste of *Nannochloropsis* sp. J Biobased Mater Bioenergy.

[CR11] Chevalier D, Le Bail A, Ghoul M (2001). Effects of high pressure treatment (100–200 MPa) at low temperature on turbot (*Scophthalmus*
*maximus*) muscle. Food Res Int.

[CR12] Darley WM, Volcani BE (1969). Role of silicon in diatom me-tabolism. A silicon requirement for deoxyribonucleic acid synthesis in the diatom *Cylindrotheca*
*fusiformis* Reimann and Lewin. Exp Cell Res.

[CR13] De Carvalho Neto RG, Da Silva Do Nascimento JG, Costa MC, Lopes AC, Abdala Neto EF, Rossas Mota Filho C, Dos Santos AB (2014) Microalgae harvesting and cell disruption: a preliminary evaluation of the technology electroflotation by alternating current. Water Sci Technol 70:315–32010.2166/wst.2014.220 10.2166/wst.2014.220 25051479

[CR14] De Oliveira CF, Giordani D, Gurak PD, Cladera-Olivera F, Marczak LDF (2015). Extraction of pectin from passion fruit peel using moderate electric field and conventional heating extraction methods. Innov Food Sci Emerg Technol.

[CR15] de Souza F, Silva AP, Costa MC, Colzi Lopes A, Fares Abdala Neto E, CarrháLeitão R, Mota CR, Bezerra dos Santos A (2014). Comparison of pretreatment methods for total lipids extraction from mixed microalgae. Renew Energy.

[CR16] de Souza MP, Hoeltz M, Gressler PD, Benitez LB, Schneider RCS (2019). Potential of microalgal bioproducts: general perspectives and main challenges. Waste Biomass Valor.

[CR17] Eing C, Goettel M, Straessner R, Gusbeth C, Frey W (2013). Pulsed electric field treatment of microalgae - benefits for microalgae biomass processing. IEEE Trans Plasma Sci.

[CR18] Ferreira-Santos P, Genisheva Z, Pereira RN, Teixeira JA, Rocha CMR (2019). Moderate electric fields as a potential tool for sustainable recovery of phenolic compounds from *Pinus*
*pinaster* b ark. ACS Sustain Chem Eng.

[CR19] Folch J, Lees M, Sloane Stanley GH (1957). A simple method for the isolation and purification of total lipides from animal tissues. J Biol Chem.

[CR20] Gallego R, Bueno M, Chourio AM, Ibáñez E, Saldaña MDA, Herrero M (2021) Use of high and ultra-high pressure based-processes for the effective recovery of bioactive compounds from *Nannochloropsis oceanica* microalgae. J Supercrit Fluids 167. 10.1016/j.supflu.2020.105039

[CR21] Gavahian M, Chu YH, Sastry S (2018). Extraction from Food and natural products by moderate electric field: mechanisms, benefits, and potential industrial applications. Compr Rev Food Sci Food Saf.

[CR22] Geada P, Rodrigues R, Loureiro L, Pereira R, Fernandes B, Teixeira JA, Vasconcelos V, Vicente AA (2018). Electrotechnologies applied to microalgal biotechnology – applications, techniques and future trends. Renew Sustain Energy Rev.

[CR23] GPS Safety Summary (2015). Global Product Strategy ( GPS ) safety summary. Hell Pertoleum.

[CR24] Hǎdǎruga DI, Ünlüsayin M, Gruia AT, Birǎu C, Rusu G, Hǎdǎruga NG (2016). Thermal and oxidative stability of Atlantic salmon oil (*Salmo*
*salar* L.) and complexation with β-cyclodextrin. Beilstein J Org Chem.

[CR25] Halim R, Papachristou I, Kubisch C, Nazarova N, Wüstner R, Steinbach D, Chen GQ, Deng H, Frey W, Posten C, Silve A (2021). Hypotonic osmotic shock treatment to enhance lipid and protein recoveries from concentrated saltwater *Nannochloropsis* slurries. Fuel.

[CR26] Hara A, Radin NS (1978). Lipid extraction of tissues with a low toxicity solvent. Anal Biochem.

[CR27] Herrero M, Ibáñez E (2015). Green processes and sustainability: an overview on the extraction of high added-value products from seaweeds and microalgae. J Supercrit Fluids.

[CR28] Islam MA, Brown RJ, O’Hara I, Kent M, Heimann K (2014). Effect of temperature and moisture on high pressure lipid/oil extraction from microalgae. Energy Convers Manag.

[CR29] Jaeschke DP, Menegol T, Rech R, Mercali GD, Marczak LDF (2016). Carotenoid and lipid extraction from *Heterochlorella*
*luteoviridis* using moderate electric field and ethanol. Process Biochem.

[CR30] Jiménez Callejón MJ, Robles Medina A, González Moreno PA, Esteban Cerdán L, OrtaGuillén S, Molina Grima E (2020). Simultaneous extraction and fractionation of lipids from the microalga *Nannochloropsis* sp. for the production of EPA-rich polar lipid concentrates. J Appl Phycol.

[CR31] Joannes C, Sipaut CS, Dayou J, Yasir SM, Mansa RF (2015) Review paper on cell membrane electroporation of microalgae using electric field treatment method for microalgae lipid extraction. IOP Conf Ser Mater Sci Eng 78. 10.1088/1757-899X/78/1/012034

[CR32] Joshi DR, Adhikari N (2019) An overview on common organic solvents and their toxicity. J Pharm Res Int 1–18. 10.9734/jpri/2019/v28i330203

[CR33] Khan SA, Aslam R, Makroo HA (2018). High pressure extraction and its application in the extraction of bio-active compounds: a review. J Food Process Eng.

[CR34] Kojima Y, Shimizu A (2022) Effect of high hydrostatic pressure treatment with room-temperature ionic liquid 1-ethyl-3-methylimidazolium acetate - dimethyl sulfoxide mixture on lipid extraction from *Chlorella vulgaris*. High Press Res 1–16. 10.1080/08957959.2022.2044032

[CR35] Ma XN, Chen TP, Yang B, Liu J, Chen F (2016) Lipid production from *Nannochloropsis*. Mar Drugs 14. 10.3390/md14040061 10.3390/md14040061 PMC4849066 27023568

[CR36] Monteiro A, Paquincha D, Martins F, Queirós RP, Saraiva JA, Švarc-Gajić J, Nastić N, Delerue-Matos C, Carvalho AP (2018). Liquid by-products from fish canning industry as sustainable sources of ω3 lipids. J Environ Manage.

[CR37] Pagels F, Pereira RN, Amaro HM, Vasconcelos V, Guedes AC, Vicente AA (2021). Continuous pressurized extraction versus electric fields-assisted extraction of cyanobacterial pigments. J Biotechnol.

[CR38] Parniakov O, Barba FJ, Grimi N, Marchal L, Jubeau S, Lebovka N, Vorobiev E (2015). Pulsed electric field assisted extraction of nutritionally valuable compounds from microalgae *Nannochloropsis* spp. using the binary mixture of organic solvents and water. Innov Food Sci Emerg Technol.

[CR39] Peltomaa E, Johnson MD, Taipale SJ (2018). Marine cryptophytes are great sources of EPA and DHA. Mar Drugs.

[CR40] Pieber S, Schober S, Mittelbach M (2012). Pressurized fluid extraction of polyunsaturated fatty acids from the microalga *Nannochloropsis*
*oculata*. Biomass Bioenerg.

[CR41] Pinela J, Prieto MA, Barros L, Carvalho AM, Oliveira MBPP, Saraiva JA, Ferreira ICFR (2018). Cold extraction of phenolic compounds from watercress by high hydrostatic pressure: process modelling and optimization. Sep Purif Technol.

[CR42] Postma PR, Pataro G, Capitoli M, Barbosa MJ, Wijffels RH, Eppink MHM, Olivieri G, Ferrari G (2016). Selective extraction of intracellular components from the microalga *Chlorella*
*vulgaris* by combined pulsed electric field-temperature treatment. Bioresour Technol.

[CR43] Prat D, Wells A, Hayler J, Sneddon H, McElroy CR, Abou-Shehada S, Dunn PJ (2015). CHEM21 selection guide of classical- and less classical-solvents. Green Chem.

[CR44] Ramesh Kumar B, Deviram G, Mathimani T, Duc PA, Pugazhendhi A (2019). Microalgae as rich source of polyunsaturated fatty acids. Biocatal Agric Biotechnol.

[CR45] Samarasinghe N (2012) Effect of high pressure homogenization on aqueous phase solvent extraction of lipids from *Nannochloris oculata* microalgae. J Energy Nat Resour 1:1. 10.11648/j.jenr.20120101.11

[CR46] Sequeira-Munoz A, Chevalier D, LeBail A, Ramaswamy HS, Simpson BK (2006). Physicochemical changes induced in carp (*Cyprinus*
*carpio*) fillets by high pressure processing at low temperature. Innov Food Sci Emerg Technol.

[CR47] Sneddon H (2017) Solvents – solvent guides, guides for chromatography, common reactions, work-ups etc. GSK. https://www.cersuschem.ufscar.br/documentos/sneddon-cersuschem-short-course-2nd-session

[CR48] Snyder LR (1978). Classification off the solvent properties of common liquids. J Chromatogr Sci.

[CR49] Sousa S, Freitas AC, Gomes AM, Carvalho AP (2022). Modulated stress to balance *Nannochloropsis*
*oculata* growth and eicosapentaenoic acid production. Appl Microbiol Biotechnol.

[CR50] Sun W, Li J, Ramaswamy HS, Yu Y, Wang C, Zhu S (2018). Adiabatic compression heating of selected organic solvents under high pressure processing. High Press Res.

[CR51] Teo CL, Idris A (2014). Enhancing the various solvent extraction method via microwave irradiation for extraction of lipids from marine microalgae in biodiesel production. Bioresour Technol.

[CR52] Udayan A, Kathiresan S, Arumugam M (2018). Kinetin and gibberellic acid (GA3) act synergistically to produce high value polyunsaturated fatty acids in *Nannochloropsis*
*oceanica* CASA CC201. Algal Res.

[CR53] Wan Mahmood WMA, Theodoropoulos C, Gonzalez-Miquel M (2017). Enhanced microalgal lipid extraction using bio-based solvents for sustainable biofuel production. Green Chem.

[CR54] Xu J, Zhao F, Su X (2021). Direct extraction of lipids from wet microalgae slurries by super-high hydrostatic pressure. Algal Res.

[CR55] Zhao Y, Jiang Y, Ding Y, Wang D, Deng Y (2019) High hydrostatic pressure-assisted extraction of high-molecular-weight melanoidins from black garlic: composition, structure, and bioactive properties. J Food Qual 2019. 10.1155/2019/1682749

